# Functionalized Ultrasmall Iron Oxide Nanoparticles for *T*_1_-Weighted Magnetic Resonance Imaging of Tumor Hypoxia

**DOI:** 10.3390/molecules27206929

**Published:** 2022-10-15

**Authors:** Lei Yang, Mohammad Javad Afshari, Jianxian Ge, Dandan Kou, Lei Chen, Dandan Zhou, Cang Li, Shuwang Wu, Leshuai Zhang, Jianfeng Zeng, Jian Zhong, Roland H. Stauber, Mingyuan Gao

**Affiliations:** 1 College of Food Science & Technology, Shanghai Ocean University, Shanghai 201306, China; 2 Center for Molecular Imaging and Nuclear Medicine, State Key Laboratory of Radiation Medicine and Protection, School for Radiological and Interdisciplinary Sciences (RAD-X), Collaborative Innovation Center of Radiological Medicine of Jiangsu Higher Education Institutions, Soochow University, Suzhou 215123, China; 3 Department of Nanobiomedicine, ENT, University Medical Center of Mainz, Langenbeckstr. 1, 55101 Mainz, Germany

**Keywords:** tumor hypoxia, ultrasmall iron oxide nanoparticles, magnetic resonance imaging, metronidazole

## Abstract

Hypoxia is a common biological condition in many malignant solid tumors that plays an imperative role in regulating tumor growth and impacting the treatment’s therapeutic effect. Therefore, the hypoxia assessment is of great significance in predicting tumor development and evaluating its prognosis. Among the plenty of existing tumor diagnosis techniques, magnetic resonance imaging (MRI) offers certain distinctive features, such as being free of ionizing radiation and providing images with a high spatial resolution. In this study, we develop a fluorescent traceable and hypoxia-sensitive *T*_1_-weighted MRI probe (Fe_3_O_4_-Met-Cy5.5) via conjugating notable hypoxia-sensitive metronidazole moiety and Cy5.5 dye with ultrasmall iron oxide (Fe_3_O_4_) nanoparticles. The results of in vitro and in vivo experiments show that Fe_3_O_4_-Met-Cy5.5 has excellent performance in relaxivity, biocompatibility, and hypoxia specificity. More importantly, the obvious signal enhancement in hypoxic areas indicates that the probe has great feasibility for sensing tumor hypoxia via *T*_1_-weighted MRI. These promising results may unlock the potential of Fe_3_O_4_ nanoparticles as *T*_1_-weighted contrast agents for the development of clinical hypoxia probes.

## 1. Introduction

The tumor microenvironment (TME), closely related to the tumor growth and metastasis [1], is often a starting point for the study of tumor development and pathological processes. During the expansion of a tumor, the tumorous tissues located far from the blood vessels might encounter a state of oxygen deficiency (i.e., hypoxia) as a consequence of the imbalance between the oxygen supply capacity and the consumption rate [2,3]. Hypoxia is an important characteristic of many pathological parameters (low pH, abnormal expression of enzymes) in TME, which greatly affects the prognosis and treatment of tumors [4,5,6]. The incidence of hypoxia in most advanced solid tumors and its capability to activate hypoxia-inducible factor-1α (HIF-1α) have been previously confirmed [7,8]. The factor can promote the invasion and metastasis of tumor cells, as well as a greater tolerance to radio- and chemotherapy [9,10]. Therefore, predicting tumor hypoxia and visualizing its distribution is of great significance for the formulation of tumor treatment protocols and the evaluation of prognosis.

Compared with the traditional oxygen electrode method [11,12], the molecular imaging technique, which can detect tumor hypoxia, is becoming more prevalent recently for its non-invasive and real-time imaging characteristics [13,14,15]. In particular, optical imaging and nuclear medicine imaging have contributed a lot to this research. In optical imaging, hypoxia fluorescent probes mostly connect dye molecules with sensitive moieties, such as nitroimidazole and azobenzene, to quench the original fluorescence until the structure of the sensitive moieties is changed by related reductase under hypoxic conditions [16]. Apart from these probes, it has been found that some phosphorescence dyes, such as the ruthenium(II) and iridium(III) complexes, can also achieve the accurate sensing of O_2_ content, according to the quenching degree of phosphorescence under different oxygen concentrations [17,18,19]. For example, our team previously reported a ratiometric O_2_ sensing probe, which innovatively encapsulated the common hydrophobic oxygen-sensitive iridium(III) complex into β-cyclodextrin and then combined it with the oxygen-insensitive Cyanine7 dye [20]. This kind of design not only improves the water solubility and oxygen sensing sensitivity of the iridium(III) complex, but also successfully realizes the rapid and quantitative visualization of hypoxic regions in vivo. Although optical imaging has superiority in resolution and sensitivity, its spatial resolution and signal intensity declines significantly with the increase of tissue depth, thus leading to limitations in deeper tissue imaging of tumors.

However, nuclear medicine imaging is free of the aforementioned limitations and displays a greater advantage in clinical application. The most deserved to be mentioned is ^18^F-fluoromisonidazole (^18^F-FMISO), which is widely used in clinical hypoxic research. It can evaluate the degree of tumor hypoxia, according to the relative signal enhancement caused by the selective accumulation in hypoxic cells [21]. Then, based on the mechanism of ^18^F-FMISO, the researchers developed a series of improved probes, such as ^18^F-fluoroazomycinarabinofuranoside (^18^F-FAZA) and ^18^F-3-fluoro-2-(4-((2-nitro-1H-imidazol-1-yl)methyl)-1H-1,2,3-triazol-1-yl)propan-1-ol (^18^F-HX4)) [22,23], which not only increase the water solubility, but also enhance the clearance rate and signal contrast. Despite this, nuclear medicine imaging is limited by low spatial resolution, thus leading to a lack of clarity in the distribution of tumor hypoxia.

Compared with the aforementioned methods, magnetic resonance imaging (MRI) has a better promising future in clearly depicting the hypoxia distribution because of its high spatial resolution and lack of limitation in tissue depth [24]. At present, Fe_3_O_4_ nanoparticles have been developed as an alternative MRI contrast agent with excellent magnetic properties and biocompatibility [25] and have gradually become an outstanding carrier for the construction of tumor hypoxia probes in recent years. For instance, Filippi et al. developed a hypoxia-specific *T*_2_-weighted MRI probe by conjugating 10 nm-sized Fe_3_O_4_ nanoparticles with the oxygen-sensitive metronidazole ligands and demonstrated its capability of selective accumulation into the hypoxic two-dimensional (2D) and three-dimensional (3D) cell models [26]. In addition, Zhou et al. constructed a hypoxia-triggered *T*_2_-weighted MRI probe by simultaneously modifying nitroimidazole and cysteine to the surface of Fe_3_O_4_ nanoparticles [27]. Under the hypoxic environment, the bioreductions of the nitro group can subsequently form reductive adducts with the thiol group of cysteine on Fe_3_O_4_ nanoparticles, thus cross-linking the nanoparticles to form larger assemblies and amplifying the *T*_2_-weighted MRI signal for the tumor interior region. Although these Fe_3_O_4_-based hypoxia probes exhibited splendid hypoxia selectivity, this mode highlights the lesion areas by a signal decline (darker images), which has been reported to affect the identification of tumors from internal bleeding, calcification, and metal deposition [28,29]. On the contrary, *T*_1_-weighted MRI is a way to brighten the regions of interest by an increase in signal (using *T*_1_-weighted images) intensity, which is intrinsically more sensitive than a decrease in signal (using *T*_2_-weighted images) intensity presented by *T*_2_-weighted MRI for reducing the impact on diagnostic accuracy. From this point of view, *T*_1_-weighted magnetic resonance imaging will have a better application prospect in the field of tumor hypoxia detection.

**Scheme 1:**
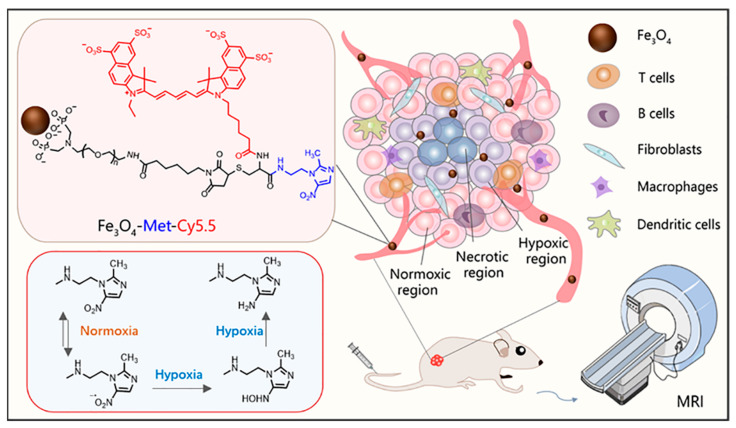
The illustration of the Fe_3_O_4_-Met-Cy5.5 nanoprobe for in vivo *T*_1_-weighted MRI and the mechanism of metronidazole trapped in hypoxic regions.

According to the report, Fe_3_O_4_ nanoparticles can also be used as *T*_1_-weighted contrast agents when the core diameter is less than 5 nm [30,31,32,33]. Herein, to realize the *T*_1_-weighted MRI of tumor hypoxia, as shown in Scheme 1, ultrasmall PEGylated Fe_3_O_4_ nanoparticles were synthesized and further conjugated with metronidazole and Cy5.5 fluorescent dye to construct a *T*_1_-weighted MRI nanoprobe (Fe_3_O_4_-Met-Cy5.5). Firstly, the nanoprobes reach the tumor tissue through the endothelial permeability and retention effect (EPR). Then, the nitro groups on the surface of nanoprobes will undergo a series of reduction reactions to form amino compounds under the action of reductase in hypoxic cells [34,35]. Finally, the compounds would eventually form bonds with macromolecular substances existing in the hypoxic regions [36], producing specific retention in hypoxia regions. Both the cellular evaluation and tumor imaging studies have proven that the Fe_3_O_4_-Met-Cy5.5 probe has excellent hypoxia sensitivity and can achieve a great *T*_1_-weighted MRI of hypoxic tumors in vivo.

## 2. Materials and Methods

### 2.1. Materials

Iron(III) acetylacetonate (Fe(acac)_3_), oleic acid, oleylamine, cyclohexane, N-hydroxysuccinimide (NHS), D-cysteine hydrochloride monohydrate (Cys), and Cy5.5-NHS ester were purchased from Aladdin. Tetrahydrofuran (THF) and acetone was purchased from Sinopharm Chemical Reagent Co., LTD., Shanghai, China. The 1-ethyl-3-(3-dimethylaminopropyl) carbodiimide hydrochloride (EDC) was purchased from Energy Chemical. Polyethylene glycol (*M*_w_ ≈ 2000 Da) with a diphosphate group at one end and a maleimide group at the other end, denoted as DP-PEG-Mal, was provided by Suzhou Xinying Biomedical Technology Co., LTD., Suzhou, China. Boc-Cys(Trt)-OH was purchased from Shanghai Maclin Biochemical Technology Co., LTD., Shanghai, China. The 1-(2-Aminoethyl)-2-methyl-5-nitroimidazoledihydrochloride was purchased from Acros Organics. Triethylsilane was purchased from TCI. Dulbecco’s modified eagle medium (DMEM) and fetal bovine serum (FBS) were purchased from HyClone. Hoechst 33342 reagent was purchased from Beyotime Biotech. HIF-1α mouse monoclonal antibody was purchased from Affinity.

### 2.2. Cells and Animals

Human breast cancer cells (MCF-7) were cultured in high glucose DMEM medium supplemented with 10% fetal bovine serum and 1% penicillin-streptomycin solution. The normoxic MCF-7 cells were cultured under the condition of 37 °C, 21% O_2_, 5% CO_2_, and 95% humid air in a carbon dioxide incubator. The hypoxic cells were cultured in a three-gas incubator with low oxygen concentration. SPF nude mice (female, 4–5 weeks, weight of 18~20 g) were purchased from Changzhou Cavens Experimental Animal Co., Ltd., Changzhou, China. All animal experiments were approved by the Laboratory Animal Center of Soochow University. The subcutaneous tumor models were established by injecting MCF-7 cells (4 × 10^6^ each) into the dorsal area near the right hind limb of each mouse.

### 2.3. Synthesis of Hypoxia-Sensitive Ligand (Met)

Boc-Cys(Trt)-OH (231.8 mg, 0.5 mmol), EDC (134.2 mg, 0.7 mmol), and NHS (135.8 mg, 1.18 mmol) were dissolved in CH_2_Cl_2_ (5 mL), stirring for 20 min at room temperature under the atmosphere of nitrogen. The mixture solution of dimethylformamide (DMF) and N-diisopropylethylamine (DIPEA) containing 1-(2-Aminoethyl)-2-methyl-5-nitroimidazoledihydrochloride (170 mg, 0.65 mmol) was slowly injected into the above solution by a syringe, under the condition of ice bath, and stirred at room temperature overnight. After the reaction, CH_2_Cl_2_ and DMF were evaporated by decompression rotation and freeze-dried to fully remove the liquid from the system. The yellow solid was extracted with saturated sodium bicarbonate solution three times. The organic phase was collected and dried with anhydrous sodium sulfate. After filtering and evaporating, the compound BM was further purified by Elite P3500 semi-preparative liquid chromatographic system.

The purified product BM (120 mg, 0.195 mmol) was dissolved in CH_2_Cl_2_ and dripped with trifluoroacetic acid (TFA) and triethylsilane. The final product, Met, was purified by Elite P3500 semi-preparative liquid chromatographic system after the solvent was removed under reduced pressure.

### 2.4. Synthesis of Ultrasmall Fe_3_O_4_ Nanoparticles

Ultrasmall iron oxide nanoparticles (Fe_3_O_4_) were prepared according to a previous report on a flow synthesis system [37]. Typically, Fe (acac)_3_ (7.1 g, 20 mmol), oleic acid (38.7 g, 137 mmol), and oleylamine (36.6 g, 137 mmol) were dissolved in 0.9 L methylbenzene to prepare a flow reaction solution. The flowing reaction liquid was pumped into the tubular reactor by a high-pressure constant current pump. And the flow rate, temperature, and residence time were 30 mL min^−1^, 270 °C, and 6 min. After that, the solution was cooled to room temperature and collected in sample bottles. Then, the crude product was precipitated with acetone and dissolved with cyclohexane for two cycles. Finally, the precipitation was redispersed in cyclohexane for further experiments.

### 2.5. Ligand Exchange to Prepare PEGylated Fe_3_O_4_-Mal Nanoparticles

A total of 100 mg DP-PEG-Mal was dissolved in 4 mL THF containing 10 mg Fe_3_O_4_ nanoparticles and stirred at 60 °C for 24 h. Then, the solution was precipitated by adding cyclohexane and dissolved in THF afterward. After 2 cycles of purification, the precipitation was dried under a vacuum and dispersed in water. The aqueous solution was then ultrafiltrated four times with a 30 kDa MWCO centrifugal filter.

### 2.6. Preparation of Fe_3_O_4_-Met-Cy5.5 and Fe_3_O_4_-Cys-Cy5.5 Nanoprobes

Met (0.25 mg, 0.9 μmol) dissolving in DMSO or Cys (0.16 mg, 0.9 μmol) dissolving in water were mixed, respectively, with the aqueous solution of tris(2-carboxyethyl) phosphine (80 μL, 4 μmol). After being adjusted to neutrality by HEPES buffer (pH = 7.4, 1 M), the mixture was added to Fe_3_O_4_-Mal (containing 2.5 mg Fe) and oscillated for 12 h to obtain Fe_3_O_4_-Met or Fe_3_O_4_-Cys nanoprobes.

Cy5.5-NHS (0.13 mg, 0.18 μmol), dissolved in DMSO (65 μL), was added to Fe_3_O_4_-Met or Fe_3_O_4_-Cys (containing 2.5 mg Fe) and stirred for 12 h. The final product was dialyzed with Dialysis bags (MWCO: 12,000-14,000 Da) for 3 days and concentrated by a 30 kDa MWCO centrifugal filter.

### 2.7. Characterizations

Proton nuclear magnetic resonance (^1^H NMR) spectra was obtained from Bruker Advance NEO 400 MHz. The morphology and size of the nanoparticles were taken with a high-resolution transmission electron microscope (Tenia G2 F20, FEI company, Hillsboro, CA, USA) at an acceleration voltage of 200 kV. The iron concentration was obtained by the 1,10-phenanthroline spectrophotometric method, after the resulting nanoparticles were eroded with the concentrated hydrochloric acid. The hydrodynamic size and zeta potential of nanoprobes were measured by Malvern Zetasizer Nano ZS90. Ultraviolet–visible absorption spectra were performed on the Persee dual-beam UV–Vis spectrophotometer. Fluorescence spectra were recorded by a steady-state/lifetime spectrofluorometer (FLS 980, Edinburgh Instruments, Livingston, Scotland). The relaxivity measurements were obtained by a 3 T animal MRI scanner (MRS 3000, MR Solution, Guildford, UK).

## 3. Results and Discussion

### 3.1. Synthesis and Characterization of Hypoxia-Sensitive Ligand

Due to the unique hypoxia-sensitive characteristics, metronidazole derivatives have been commonly used for hypoxia-selective prodrugs and imaging probes [38,39,40]. Herein, through an amidation reaction of Boc-Cys(Trt)-OH and metronidazole, followed by a deprotection reaction, 2-amino-3-mercapto-N-(2-(2-methyl-5-nitro-1H-imidazol-1-yl)ethyl)propanamide (Met)) was achieved as the hypoxia-sensitive ligand. The detailed synthesis route, ^1^H NMR, and mass spectrum of the resulting product are given in the Supporting Information (Scheme S1, Figures S1 and S2, respectively). Moreover, Cys which lacks specificity toward hypoxia, was used to serve as the control ligand.

### 3.2. Construction and Characterization of Hypoxia-Sensitive MRI Nanoprobes

**Figure 1:**
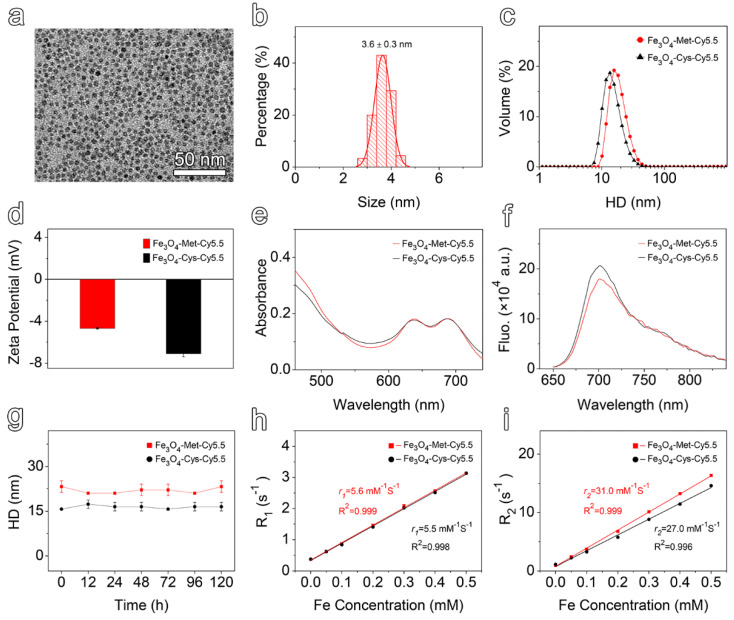
(**a**) TEM image and (**b**) size distribution of PEGylated Fe_3_O_4_-Mal nanoparticles. (**c**) Hydrodynamic size distribution and (**d**) zeta potential values of Fe_3_O_4_-Met-Cy5.5 and Fe_3_O_4_-Cys-Cy5.5. (**e**) Ultraviolet–visible absorption spectra of Fe_3_O_4_-Met-Cy5.5 and Fe_3_O_4_-Cys-Cy5.5, with a Fe concentration of 50 μg mL^−1^. (**f**) Fluorescence spectra of Fe_3_O_4_-Met-Cy5.5 and Fe_3_O_4_-Cys-Cy5.5, with a Fe concentration of 50 μg mL^−1^ acquired at 705 nm upon excitation at 635 nm. (**g**) The hydrodynamic size variation of Fe_3_O_4_-Met-Cy5.5 and Fe_3_O_4_-Cys-Cy5.5 incubated in PBS for 120 h. Linear regression fitting of the longitudinal (**h**) and transverse (**i**) relaxivities of Fe_3_O_4_-Met-Cy5.5 and Fe_3_O_4_-Cys-Cy5.5 for extracting *r*_1_ and *r*_2_.

The transmission electron microscopy (TEM) images and the corresponding particle size distribution of ultrasmall Fe_3_O_4_ nanoparticles were provided in Figure S3a in the Supporting Information. Polyethylene glycol (PEG) polymers, bearing a diphosphate group at one end and maleimide at the other, were employed to render the prepared nanoparticles hydrophilic by replacing the native organic ligands of the hydrophobic Fe_3_O_4_ nanoparticles. As shown in Figure 1a,b, the resulting maleimide functionalized nanoparticles (Fe_3_O_4_-Mal) exhibited a uniform spherical shape, with a mean size of 3.6 ± 0.3 nm. Subsequently, the Met or Cys ligands was conjugated to the Fe_3_O_4_-Mal nanoparticles by a click reaction taking place between the maleimide groups of nanoparticles and the thiol moieties of the ligands. Finally, the surfaces of the resulting products were further modified by Cy5.5-NHS ester to construct the final hypoxia-sensitive and non-sensitive nanoprobes, termed Fe_3_O_4_-Met-Cy5.5 and Fe_3_O_4_-Cys-Cy5.5, respectively (Scheme S2). As displayed in Figure S3b,c, the surface modification processes did not alert the morphology and size distribution of the original nanoparticles, such that spherical particles with the size of 3.7 ± 0.4 nm were prepared in both cases. The dynamic light scattering (DLS) measurement results given in Figure 1c show that both Fe_3_O_4_-Met-Cy5.5 and Fe_3_O_4_-Cys-Cy5.5 exhibit narrow hydrodynamic size distribution profiles. However, the hydrodynamic size (Figure 1c) and zeta potential value (Figure 1d) of Fe_3_O_4_-Met-Cy5.5 were slightly higher than those of Fe_3_O_4_-Cys-Cy5.5, which might be caused by the modification of the metronidazole groups. Anyway, the emergence of Cy5.5 characteristic absorption and emission peaks in the corresponding spectra (Figure 1e,f) of the resultant nanoprobes confirmed that two ligands and the dye molecules have successfully conjugated to the surface of the nanoprobes. Through ultraviolet–visible spectroscopy, the number of Cy5.5 moieties was estimated to be 4 on each nanoparticle.

To evaluate the colloidal stability, the prepared nanoprobes were incubated in water or PBS for up to 120 h, and the hydrodynamic sizes were monitored by DLS. The results shown in Figure 1g and Figure S4a exhibited negligible change, which translated into the perfect colloidal stability of the nanoprobes. Subsequently, the relaxation measurements were performed under 3 T to assess the imaging performances of the prepared nanoprobes. As shown in Figure S4b,c, increasing the concentration caused the signal intensity to increase under *T*_1_-weighted mode and decrease under *T*_2_-weighted mode, respectively. Through linear regression fitting of the experimental relaxation rates, as shown in Figure 1h, the longitudinal relaxivity (*r*_1_) of Fe_3_O_4_-Met-Cy5.5 and Fe_3_O_4_-Cys-Cy5.5 were calculated to be 5.6 mM^−1^ s^−1^ and 5.5 mM^−1^ s^−1^, respectively. Similarly, as illustrated in Figure 1i, the transverse relaxivity (*r*_2_) values of 31.0 mM^−1^ s^−1^ and 27.0 mM^−1^ s^−1^ were measured for the hypoxia sensitive and non-sensitive probes, respectively. The high *r*_1_ value and the low *r*_2_/*r*_1_ ratio suggested that both of the prepared nanoprobes exhibited great relaxation performance and can be employed as promising contrast agents for *T*_1_-weighted MRI.

### 3.3. In Vitro Specificity and Cytotoxicity of Hypoxia-Sensitive MRI Nanoprobes

**Figure 2:**
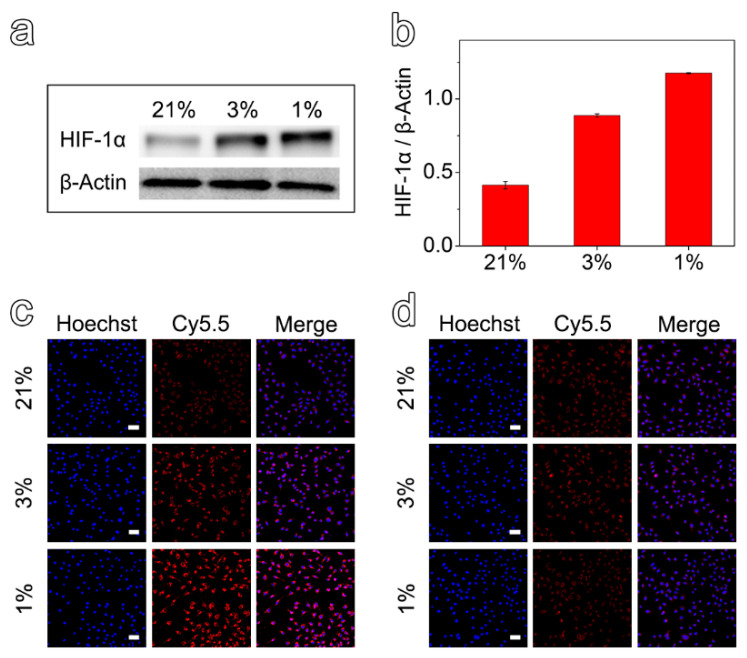
(**a**) Expression of HIF-1α protein in MCF-7 cells incubated under the oxygen concentrations of 1%, 3%, and 21% for 12 h. (**b**) Semi-quantitative analysis of relative expression of HIF-1α protein at different oxygen concentrations. Confocal fluorescence images of MCF-7 cells after incubation with Fe_3_O_4_-Met-Cy5.5 (**c**) or Fe_3_O_4_-Cys-Cy5.5 (**d**) at different oxygen concentrations (scale bar represents 50 μm).

To investigate the efficacy of the prepared nanoprobes in detecting hypoxic environments, MCF-7 cells with different hypoxic states were first obtained by incubating the cells under different oxygen concentrations for 12 h. Then, the expression of HIF-1α was evaluated by Western Blot assays. As shown in Figure 2a,b, it was verified that the cells cultured in 3% and 1% O_2_ could establish hypoxic cell models for further experiments. Anyway, the protein expression values for the cells cultured under 1% and 3% O_2_ were 2.9 and 2.2 times higher than that of 21%, respectively.

After demonstrating the different hypoxic states, the Fe_3_O_4_-Met-Cy5.5 and Fe_3_O_4_-Cys-Cy5.5 nanoprobes were incubated with hypoxic and normoxic cell models and observed by a confocal laser scanning microscope. Comparing the cells incubated with the two nanoprobes at the same hypoxia level (1% or 3% O_2_), the cellular accumulation of Fe_3_O_4_-Met-Cy5.5 was much higher than that of Fe_3_O_4_-Cys-Cy5.5 (Figure 2c,d). On the other hand, evaluating the cells incubated under three oxygen concentrations revealed that the cellular uptake of Fe_3_O_4_-Met-Cy5.5 increased in response to the decrease in the oxygen level. On the contrary, no apparent variation in cellular accumulation could be identified for the cells incubated with Fe_3_O_4_-Cys-Cy5.5. Particularly, as shown in Figure S5, the average fluorescence intensity arising from the Fe_3_O_4_-Met-Cy5.5 group under the oxygen concentrations of 1% and 3% were 2.8 and 1.6 times higher than that under 21% O_2_, respectively. It was noticed that the fluorescence enhancement of the Fe_3_O_4_-Met-Cy5.5 group was well-consistent with the increase of its HIF-1α expression under a more severe state of hypoxia (1% O_2_). To further prove that the Fe_3_O_4_-Met-Cy5.5 probe has more uptake in a hypoxic environment, the nanoprobes were incubated with cells treated with hypoxia (1% O_2_) or normoxia (21% O_2_) for 12 h, and then followed by Prussian blue staining. As shown in Figure S6, more blue substances appeared in the cells of Fe_3_O_4_-Met-Cy5.5 group under 1% O_2_. These observations further confirmed that the Fe_3_O_4_-Met-Cy5.5 probe is sensitive to hypoxia cells, especially under severe hypoxia. The main reason is the accumulation and retention effect caused by the combination of metronidazole moieties and macromolecules in the hypoxic environment.

**Figure 3:**
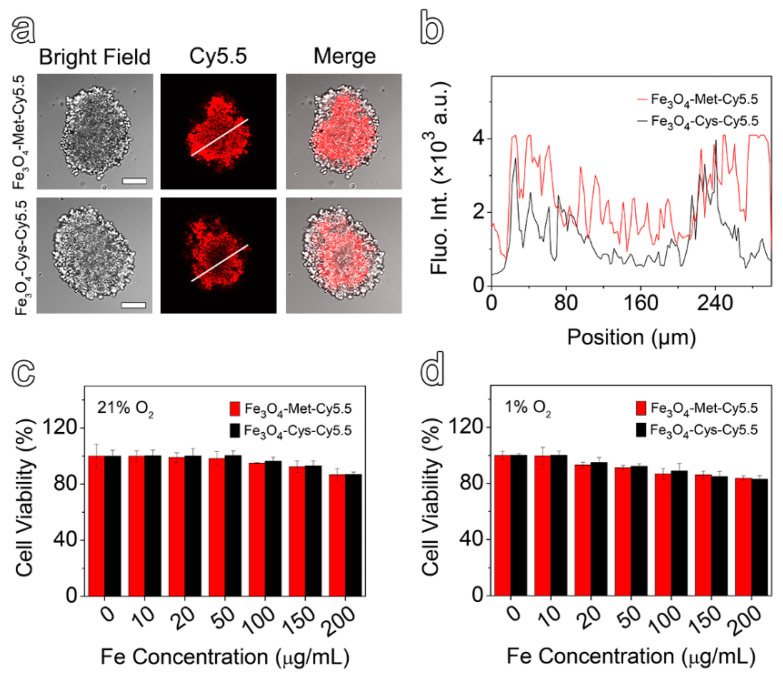
(**a**) The bright field and fluorescence images of multicellular spheres in the profile of 50 μm from the maximum cross-section (scale bar represents 100 μm). (**b**) The relative fluorescence intensity distribution, along with the white line drawn on the fluorescence images. MCF-7 cells viabilities by CCK-8 assay after incubating with varied concentrations of Fe_3_O_4_-Cys-Cy5.5 or Fe_3_O_4_-Met-Cy5.5 under 21% (**c**) and 1% O_2_ (**d**).

In comparison with the 2D cell model, the 3D multicellular spheres, which are better in line with the growth state of living cells in vivo, have been widely used in drug evaluation [41,42]. In addition, it has been reported that, for the multicellular spheres larger than 200 μm in diameter, the conditions of hypoxia and necrosis might be induced due to the lack of nutrition supply [43]. Herein, the spheres with approximate diameters of 300 μm were selected to be incubated with Fe_3_O_4_-Met-Cy5.5 and Fe_3_O_4_-Cys-Cy5.5 for 6 h. The representative slice images of multicellular spheres, obtained by a confocal laser scanning microscope, are given in Figure 3a, together with the quantified fluorescence intensity results shown in Figure 3b. It is easily noticeable that the fluorescence signal of the multicellular spheres incubated with Fe_3_O_4_-Met-Cy5.5 was stronger than that of the control group, especially in hypoxic inner areas. Furthermore, the intensity spectra close to the interior (100 to 200 μm) were integrated. It was found that the fluorescence intensity of the Fe_3_O_4_-Met-Cy5.5 group was 2.1 times higher than that of Fe_3_O_4_-Cys-Cy5.5. The results proved that the Fe_3_O_4_-Met-Cy5.5 probe also had a superior ability to be retained in the hypoxic regions of 3D multicellular spheres.

To evaluate the cytotoxicity of Fe_3_O_4_-Met-Cy5.5 and Fe_3_O_4_-Cys-Cy5.5, the standard cell counting kit 8 (CCK-8) assays were performed based on the proliferation of MCF-7 cells under 21% and 1% O_2_. As depicted in Figure 3c,d, the viability values of the cells incubated with nanoprobes containing up to 200 μg mL^−1^ of Fe were higher than 80%, regardless of the O_2_ concentration. These results suggested that both nanoprobes exhibited low cytotoxicity under both normoxic and hypoxic conditions and can be applied to in vivo imaging experiments.

### 3.4. In Vivo T_1_-Weighted MRI of Tumors

**Figure 4:**
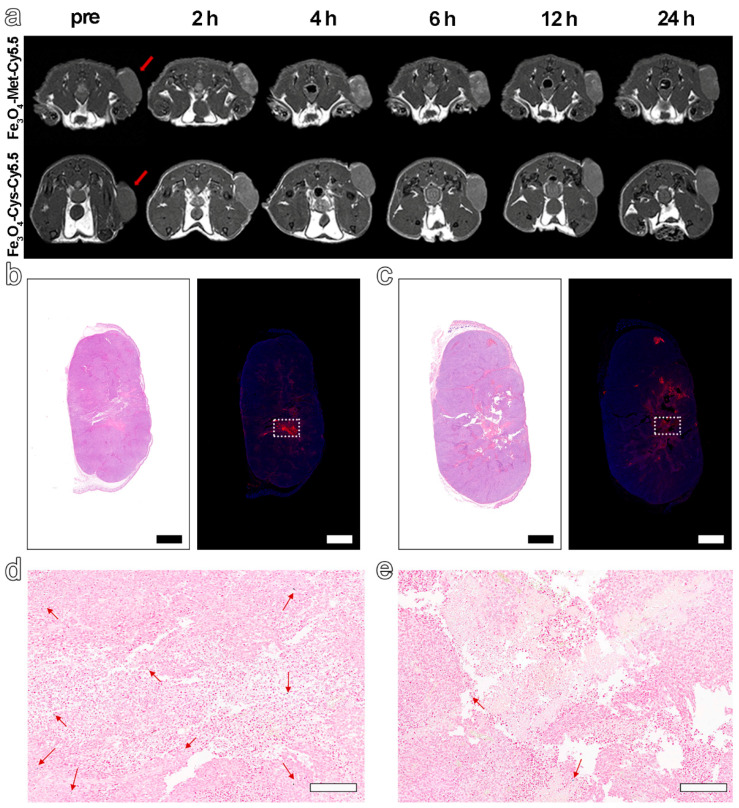
(**a**) *T*_1_-weighted MRI of tumor-bearing mice within 24 h, before and after injection with Fe_3_O_4_-Met-Cy5.5 or Fe_3_O_4_-Cys-Cy5.5. H&E staining (left) and immunofluorescence staining (right) for tumors of mice injected with Fe_3_O_4_-Met-Cy5.5 (**b**) or Fe_3_O_4_-Cys-Cy5.5 (**c**) (scale bars represent 1000 μm). Prussian blue staining of hypoxic area (dashed area on immunofluorescence staining images) in the tumors of mice treated with Fe_3_O_4_-Met-Cy5.5 (**d**) or Fe_3_O_4_-Cys-Cy5.5 (**e**) (scale bars represent 200 μm).

Based on the excellent hypoxia sensitivity *in vitro*, the nanoprobes were further intravenously injected into MCF-7 tumor-bearing mice (5.6 mg Fe kg^−1^ bodyweight) to explore the ability to detect hypoxia in vivo via MRI. The *T*_1_-weighted MR images at different time points before (pre) and post-injection (2 h, 4 h, 6 h, 12 h, 24 h) were collected by 3 T MRI apparatus. The results in Figure 4a displayed an obvious brightening trend in the signal arising from the tumor site for both groups and up to 6 h post-injection. However, from 6 to 24 h after injection, a decreasing signal tendency was identified for both groups. Furthermore, the normalized ratio of signal intensity arising from the tumor site to that of normal muscle (T/N) in Figure 4a and Figure S7 clearly exhibited that Fe_3_O_4_-Met-Cy5.5 could provide a higher contrast than that of Fe_3_O_4_-Cys-Cy5.5 (1.39 vs. 1.18 at 6 h post-injection). Anyway, the trend of the MRI signals in the two groups had statistically significant differences at 2, 4, 6, and 12 h post-injection (Figure S8a). Based on the above results, the ability of Fe_3_O_4_-Met-Cy5.5 and Fe_3_O_4_-Cys-Cy5.5 to target the tumors through the EPR effect can presumably be considered similar, due to their similar sizes and surface modifications. Therefore, this higher tumor contrast was assumed to originate from the hypoxic condition of the tumor, which might lie in the specific accumulation of metronidazole moieties. This conjecture is reasonable as the central area of the tumor site in Figure 4a, which preferred a hypoxic state, due to the limited oxygen supply capacity, and was visibly brighter than the surrounding region of the tumor treated with Fe_3_O_4_-Met-Cy5.5. To verify this assumption, the tumors were harvested and subjected to H&E and immunofluorescence staining of the HIF-1α antibody. As illustrated in Figure 4b,c, areas of tumor necrosis in H&E staining and hypoxic regions in HIF-1α staining were identified for both groups. More importantly, the distribution of the red fluorescence of HIF-1α, given in Figure 4b, was generally consistent with the brightening area of Fe_3_O_4_-Met-Cy5.5-treated group, verifying that our previous assumption was valid. In addition, Prussian blue staining of the hypoxic regions from the adjacent slices showed that both nanoprobes still had retention in the tumor regions after injection for 24 h (Figure 4d,e).

**Figure 5:**
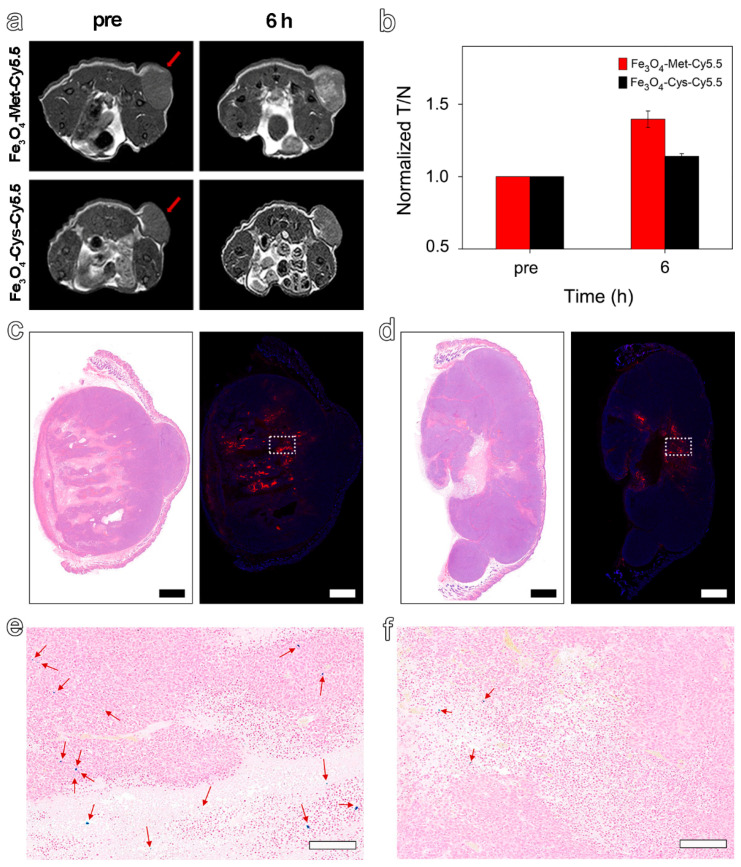
(**a**) *T*_1_-weighted MRI of tumor-bearing mice before (pre) and 6 h post-injection of Fe_3_O_4_-Met-Cy5.5 or Fe_3_O_4_-Cys-Cy5.5. (**b**) The MRI signals at the time before (pre) and after (6 h) injection of Fe_3_O_4_-Met-Cy5.5 or Fe_3_O_4_-Cys-Cy5.5. H&E staining (left) and immunofluorescence staining (right) for tumors of mice injected with Fe_3_O_4_-Met-Cy5.5 (**c**) or Fe_3_O_4_-Cys-Cy5.5 (**d**) (scale bars represent 1000 μm). Prussian blue staining of hypoxic area (dashed area on immunofluorescence staining images) in the tumors of mice treated with Fe_3_O_4_-Met-Cy5.5 (**e**) or Fe_3_O_4_-Cys-Cy5.5 (**f**) (scale bars represent 200 μm).

As the hypoxic areas in tumors may change slightly over time, the MRI was scanned again with the termination at the highest signal value (6 h) to further explore the ability of Fe_3_O_4_-Met-Cy5.5 to visualize hypoxia distribution. As expected, we found a highly brightening contrast again in the central region of the tumor treated with Fe_3_O_4_-Met-Cy5.5 (Figure 5a), and its signal value at 6 h was 1.40-fold higher than that of Pre (Figure 5b). However, the brightening effect was much weaker in the control group, and its signal value was only a 1.14-fold enhancement. As shown in Figure 5c,d, there were obvious positive areas in the center of the tumor, and the positive areas in the Fe_3_O_4_-Met-Cy5.5 group were well-consistent with the signal-enhanced regions in the MRI images at 6 h. In addition, the expression of HIF-1α in the central areas was significantly stronger than that of the surrounding less-hypoxic areas (Figure S8b). The above results indicated that the Fe_3_O_4_-Met-Cy5.5 probe can achieve *T*_1_-weighted MRI of hypoxic areas in tumors. Moreover, Prussian blue staining results demonstrated that the number of blue spots for the tumor treated with Fe_3_O_4_-Met-Cy5.5 was significantly larger than that of the control group (Figure 5e,f), further proving the higher accumulation and retention of Fe_3_O_4_-Met-Cy5.5 in hypoxic tumors. Anyway, the H&E staining (Figure S9) of both groups showed no obvious pathological symptoms of inflammation, cell edema, and necrosis in the tissues of tumor-bearing mice, indicating the biosafety of the two nanoprobes at the dosage of 5.6 mg Fe kg^−1^.

## 4. Conclusions

In conclusion, we have successfully constructed an ultrasmall Fe_3_O_4_-based *T*_1_-weighted MRI probe with outstanding relaxivity performance, biocompatibility, and hypoxia specificity. The in vitro cell experiments showed that the accumulation of Fe_3_O_4_-Met-Cy5.5 under hypoxic condition was consistent with the increase of HIF-1α expression level, which proved the excellent hypoxia sensitivity of the resultant nanoprobes. After being intravenously injected into the tumor-bearing mice model, the significantly enhanced signal contrast in the tumors’ interior regions confirmed the hypoxia sensitivity of the Fe_3_O_4_-Met-Cy5.5 in vivo. Anyway, the comparison between the brightening MRI and hypoxia immunofluorescence images further demonstrated the feasibility of the resultant nanoprobes for depicting the hypoxia area in tumors. Therefore, this work not only exhibits the potential of ultrasmall Fe_3_O_4_ in tumor diagnostic imaging as *T*_1_-weighted MRI contrast agents but also provides a valuable reference for hypoxia imaging probes in the future.

## Data Availability

Data are contained within the article or supplementary material.

## Supplementary Materials

The following supporting information can be downloaded at: https://www.mdpi.com/article/10.3390/molecules27206929/s1, Scheme S1: Synthetic route of hypoxia-sensitive ligand (Met), Scheme S2: Synthetic route of Fe_3_O_4_-Met-Cy5.5 and Fe_3_O_4_-Cys-Cy5.5, Figure S1: ^1^H NMR spectrum of hypoxia-sensitive ligand (Met), Figure S2: Mass spectrum of hypoxia-sensitive ligand (Met), Figure S3: TEM images and the size distributions of Fe_3_O_4_ (a), Fe_3_O_4_-Met-Cy5.5 (b), and Fe_3_O_4_-Cys-Cy5.5 (c) nanoparticles, Figure S4: The evaluation of colloid stability for Fe_3_O_4_-Met-Cy5.5 and Fe_3_O_4_-Cys-Cy5.5 stored in water for 120 h (a). *T*_1_-weighted (b) and *T*_2_-weighted (c) MR images of the Fe_3_O_4_-Met-Cy5.5 and Fe_3_O_4_-Cys-Cy5.5 at different Fe concentrations (0, 0.05, 0.1, 0.2, 0.3, 0.4, 0.5 mM), Figure S5: Semi-quantitative analysis of MCF-7 cells fluorescence imaging under three oxygen concentrations, Figure S6: Prussian blue staining of MCF-7cells incubated with Fe_3_O_4_-Met-Cy5.5 or Fe_3_O_4_-Cys-Cy5.5 probe at different oxygen concentrations for 12 h (scale bar represents 10 μm). Figure S7: *T*_1_-weighted MRI of two additional tumor-bearing mice for each group within 24 h, before and after injection with Fe_3_O_4_-Met-Cy5.5 or Fe_3_O_4_-Cys-Cy5.5, Figure S8: (a) The trend of MRI signal at different time points in tumors injected with Fe_3_O_4_-Met-Cy5.5 or Fe_3_O_4_-Cys-Cy5.5. (b) Semi-quantitative analysis of HIF-1α expression of the less-hypoxic and hypoxic regions in tumors treated with Fe_3_O_4_-Met-Cy5.5 or Fe_3_O_4_-Cys-Cy5.5, Figure S9: H&E staining of organ tissue (heart, liver, spleen, lung, and kidney) from mice harvested at 24 h after the injection of Fe_3_O_4_-Met-Cy5.5 or Fe_3_O_4_-Cys-Cy5.5 (scale bar represents 100 μm). Ref. [44] appears in Supplementary Materials.

## Sample Availability

Samples of the compounds are not available from the authors.
