# Supplementary material for: Functionalized Ultrasmall Iron Oxide Nanoparticles for T1-Weighted Magnetic Resonance Imaging of Tumor Hypoxia
Source: Molecules. 2022 Oct 15;27(20):6929. doi: 10.3390/molecules27206929 (PMC9610745; doi:10.3390/molecules27206929)
Supplement: Supplementary file 1 [file molecules-27-06929-s001.zip › molecules-1921283-supplementary.pdf]

molecules

MDPI

Supplementary Materials

Functionalized Ultrasmall Iron Oxide Nanoparticles for T_1-Weighted Magnetic Resonance Imaging of Tumor Hypoxia

Lei Yang [1], Mohammad Javad Afshari [2], Jianxian Ge [2], Dandan Kou [2], Lei Chen [2], Dandan Zhou [2], Cang Li [2], Shuwang Wu [2], Leshuai Zhang [2], Jianfeng Zeng [2,*], Jian Zhong [1], Roland H. Stauber [3] and Mingyuan Gao [1,2,*]

[1] College of Food Science & Technology, Shanghai Ocean University, Shanghai 201306, China
[2] Center for Molecular Imaging and Nuclear Medicine, State Key Laboratory of Radiation Medicine and Protection, School for Radiological and Interdisciplinary Sciences (RAD-X), Collaborative Innovation Center of Radiological Medicine of Jiangsu Higher Education Institutions, Soochow University, Suzhou 215123, China
[3] Department of Nanobiomedicine, ENT, University Medical Center of Mainz, Langenbeckstr. 1, 55101 Mainz, Germany
* Correspondence: jfzeng@suda.edu.cn (J.Z.); gaomy@iccas.ac.cn (M.G.)

Hydrodynamic Size, Zeta, Potential and Stability of the Nanoprobes. The Fe_3O_4-Met-Cy5.5 and Fe_3O_4-Cys-Cy5.5 were diluted to 50 μg mL^{-1} (based on Fe content) with HEPES buffer (pH=7.4, 10 mM). The hydrodynamic size and zeta potential were measured by the Malvern Zetasizer Nano ZS90. The Fe_3O_4-Met-Cy5.5 and Fe_3O_4-Cys-Cy5.5 were diluted to 50 μg mL^{-1}(based on Fe content) with PBS or ultra-pure water. The colloidal stability was assessed by measuring the hydrodynamic size of Fe_3O_4-Met-Cy5.5 and Fe_3O_4-Cys-Cy5.5 in PBS or ultra-pure water at different time points.

Iron Concentration Test. 20 μL nanoparticles solution was first digested with 200 μL concentrated hydrochloric acid. Then, 1 mL 10% hydroxylamine hydrochloride, 5 mL acetic acid buffer (pH=4.7, 1M), 2 mL 0.5% 1,10-phenanthroline solution were sequentially added. The mixture was volume up to 50 mL with ultra-pure water. The iron concentration was determined by the absorbance at 510 nm based on standard curve of iron obtained by the same method.

Determination of the Number of Cy5.5 Moieties per Fe_3O_4 Particle. The absorbance of Fe_3O_4-Met, Fe_3O_4-Cys, Fe_3O_4-Met-Cy5.5, and Fe_3O_4-Cys-Cy5.5 solution with an iron concentration of 50 μg mL^{-1} were determined by a UV-Vis spectrophotometer. The number of Cy5.5 on each Fe_3O_4 nanoparticle can be calculated by the absorbance difference at 690 nm between Fe_3O_4-Met and Fe_3O_4-Met-Cy5.5 (or Fe_3O_4-Cys and Fe_3O_4-Cys-Cy5.5) solution based on the standard curve of Cy5.5 solution.

Relaxation Properties Measurement. Fe_3O_4-Met-Cy5.5 or Fe_3O_4-Cys-Cy5.5 were diluted to 0, 0.05, 0.1, 0.2, 0.3, 0.4, 0.5 mM with ultra-pure water and sealed in tubules. The T_1- and T_2-weighted images were acquired by a 3 T animal MRI scanner. The parameters were as follows. T_1-Weighted image: repetition time (TR) = 300 ms, echo time (TE) = 11 ms, field of view (FOV) = 40 mm×40 mm, Thickness = 2 mm; T_2-Weighted image: TR =5000 ms, TE= 68 ms, FOV = 40 mm×40 mm, Thickness = 2 mm. The parameters for T_1 measurements were set as follows: TE = 11 ms and TR = 200, 500, 1000, 1500, 2000, and 5000 ms. For T_2 measurements, the parameters were set as TR = 1200 ms and TE = 20, 40, 60, 80 and 100 ms.

Western Blot Assay. The protein was collected from MCF-7 cells incubated at 1%, 3%, and 21% O_2 for 12 h. The expression of HIF-1α was measured by western blot assay as previously reported.[1]

Fluorescence Imaging of Monolayer Cells and Multicellular Spheres. The MCF-7 cell suspension was seeded to the confocal dishes (7× 10^4 cells per dish) for overnight incubation under 21% O_2. Then dishes were separately placed under 1%, 3%, and 21% O_2 for 12 h. Subsequently, the cells were treated with Fe_3O_4-Met-Cy5.5 or Fe_3O_4-Cys-Cy5.5 (containing 15 μg Fe) for 4 h and stained with Hoechst 33342 for 15 min. In the end, fluorescence imaging of monolayer cells was examined by the confocal microscope (FV1200, Olympus).

200 μL MCF-7 cells with a density of 1000 cells mL^{-1} were added into the low adsorption 96-well plate for 12 days. The multicellular spheres, nearly 300 μm in diameter, were treated with Fe_3O_4-Met-Cy5.5 or Fe_3O_4-Cys-Cy5.5 (containing 50 μg Fe) for 6 h. After washing with PBS three times, the images of multicellular spheres were acquired by the confocal microscope.

Prussian Blue Staining *In vitro*. The MCF-7 cell suspension was seeded to the 24-well plate (3× 10^4 cells per well) for overnight incubation under 21% O_2. Then dishes were separately placed under 21% and 1% O_2 for 12 h. Subsequently, the cells were treated with Fe_3O_4-Met-Cy5.5 or Fe_3O_4-Cys-Cy5.5 (containing 50 μg Fe) for 12 h under 21% and 1% O_2, respectively. Then the cells were fixed with 4% paraformaldehyde for 30 min at room temperature and treated with the mixture solution of 4% potassium ferrocyanide and 12% HCl (volume ratio was 1:1) for 1 h at room temperature.

***In Vitro* Cytotoxicity Assay.** The suspension of MCF-7 cells were seeded to 96-well plates (6000 cells per well) and cultured under 21% O_2. After removing the medium in the bottle, the prepared Fe_3O_4-Met-Cy5.5 or Fe_3O_4-Cys-Cy5.5 at different Fe concentration (0, 10, 20, 50, 100, 150, 200 μg mL^{-1}) with DMEM was added to plates respectively. Two plates of cells were incubated in 1% O_2 and the other two were in 21% O_2 for 24 h. Then 100 μL CCK8 solution was added to each well and incubated for 1 h. The cytotoxicity was required by the absorbance of the CCK8 solution at 450 nm.

The Assessment of MRI *In Vivo*. At first, nude mice bearing MCF-7 tumors were scanned by a 3 T animal MRI scanner before injection. Then the T_1-weighted MRI was performed at specific time points after the intravenous injection with nanoprobes (5.6 mg Fe kg^{-1} bodyweight). The parameters were as follows: TR = 720 ms, TE = 11 ms, FOV = 40 mm × 40 mm, Thickness = 1 mm.

Histopathology and Immunofluorescence Staining. The main organs (heart, liver, spleen, lung, and kidney) and tumor tissues of mice were extracted and cut into slices after MRI. Then the organ tissues were stained by H&E for histopathological observation. The slices of tumor tissue were subjected to H&E staining, HIF-1α staining, and Prussian blue staining respectively.

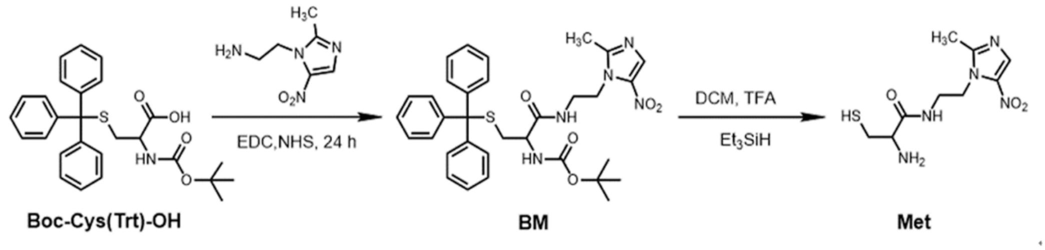

Scheme S1. Synthetic route of hypoxia-sensitive ligand (Met).

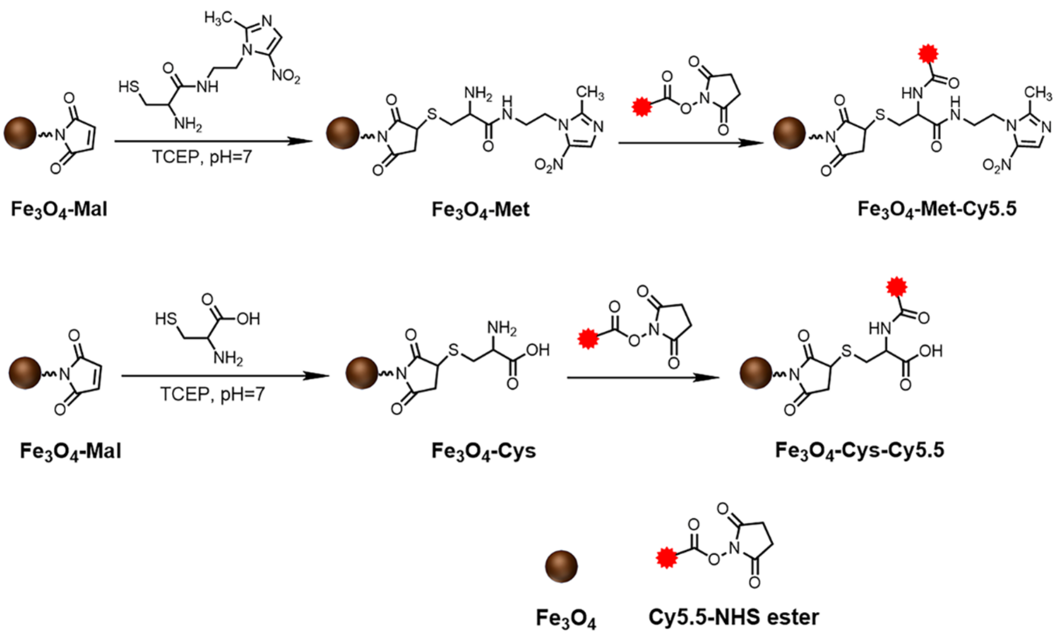

Scheme S2. Synthetic route of Fe_3O_4-Met-Cy5.5 and Fe_3O_4-Cys-Cy5.5.

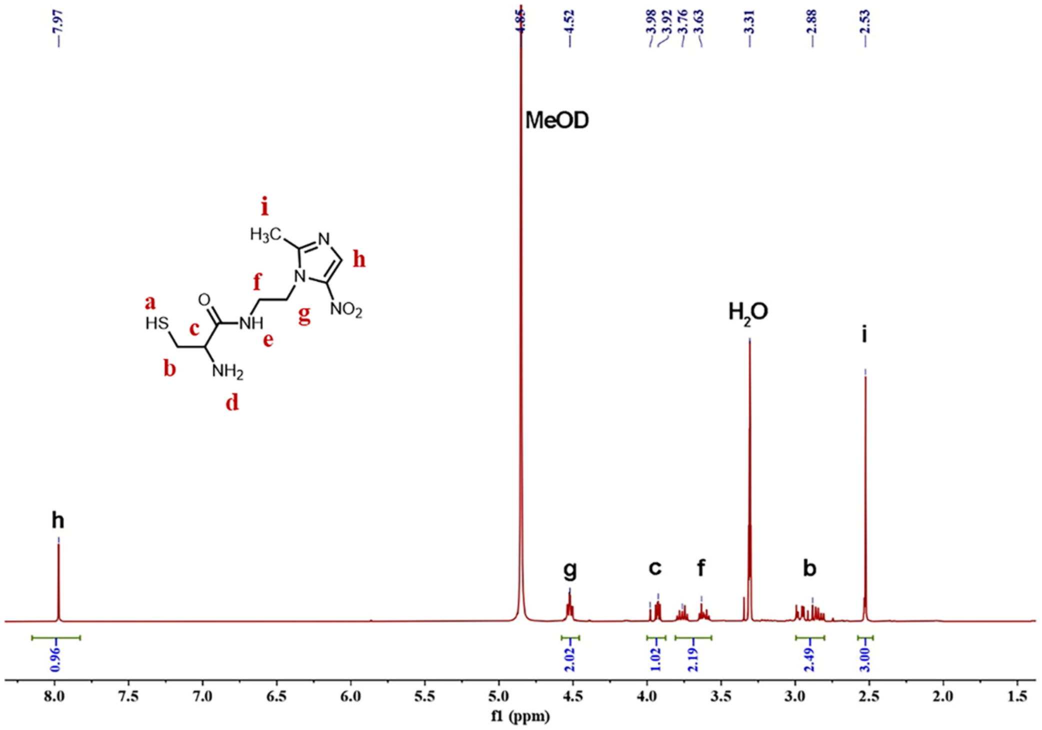

Figure S1. ^{1}H NMR spectrum of hypoxia-sensitive ligand (Met).

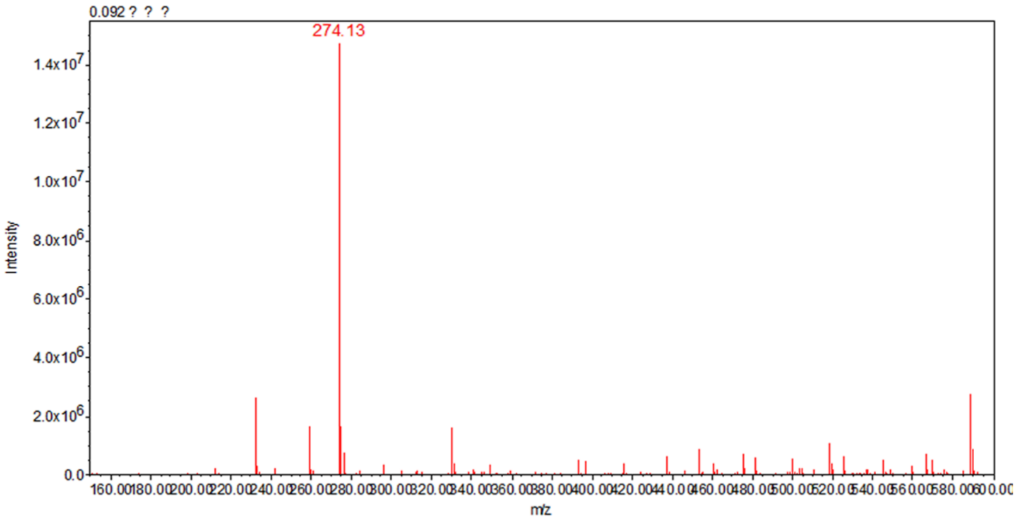

Figure S2. Mass spectrum of hypoxia-sensitive ligand (Met).

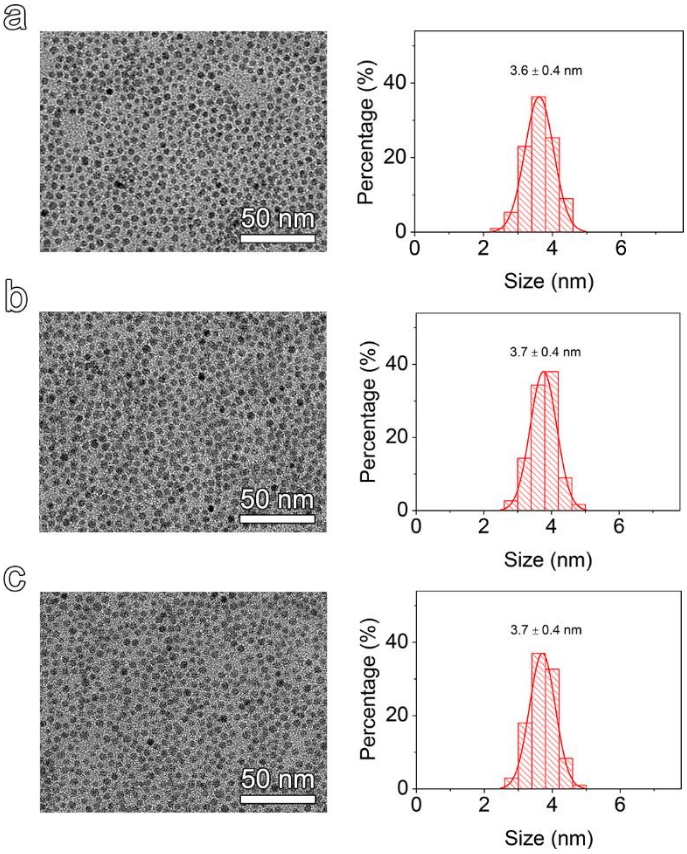

Figure S3. TEM images and the size distributions of Fe_3O_4 (**a**), Fe_3O_4-Met-Cy5.5 (**b**), and Fe_3O_4-Cys-Cy5.5 (**c**) nanoparticles.

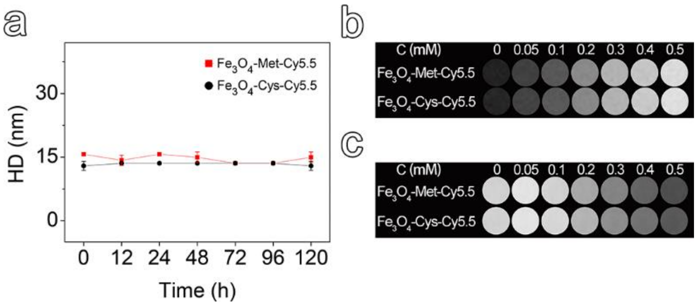

Figure S4. The evaluation of colloid stability for Fe_3O_4-Met-Cy5.5 and Fe_3O_4-Cys-Cy5.5 stored in water for 120 h (**a**). T_1-weighted (**b**) and T_2-weighted (**c**) MR images of the Fe_3O_4-Met-Cy5.5 and Fe_3O_4-Cys-Cy5.5 at different Fe concentrations (0, 0.05, 0.1, 0.2, 0.3, 0.4, 0.5 mM).

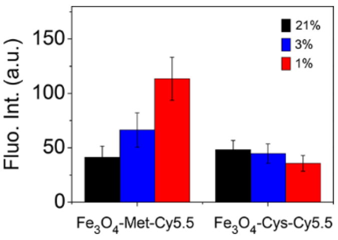

Figure S5. Semi-quantitative analysis of MCF-7 cells fluorescence imaging under three oxygen concentrations.

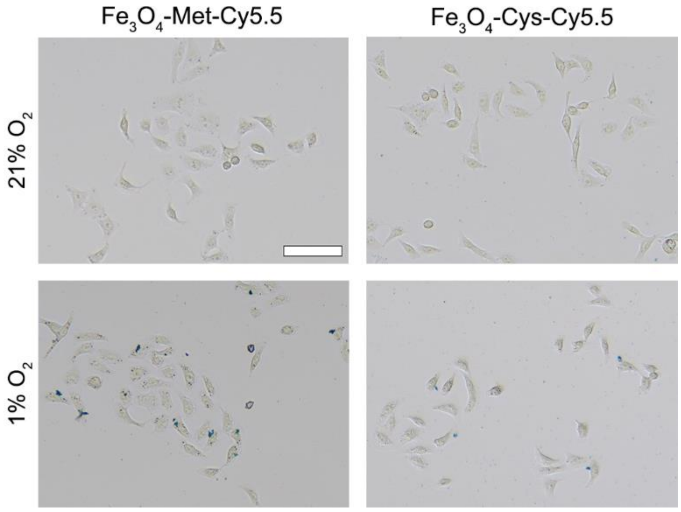

Figure S6. Prussian blue staining of MCF-7 cells incubated with Fe_3O_4-Met-Cy5.5 or Fe_3O_4-Cys-Cy5.5 probe at different oxygen concentrations for 12 h (scale bar represents 10 μm).

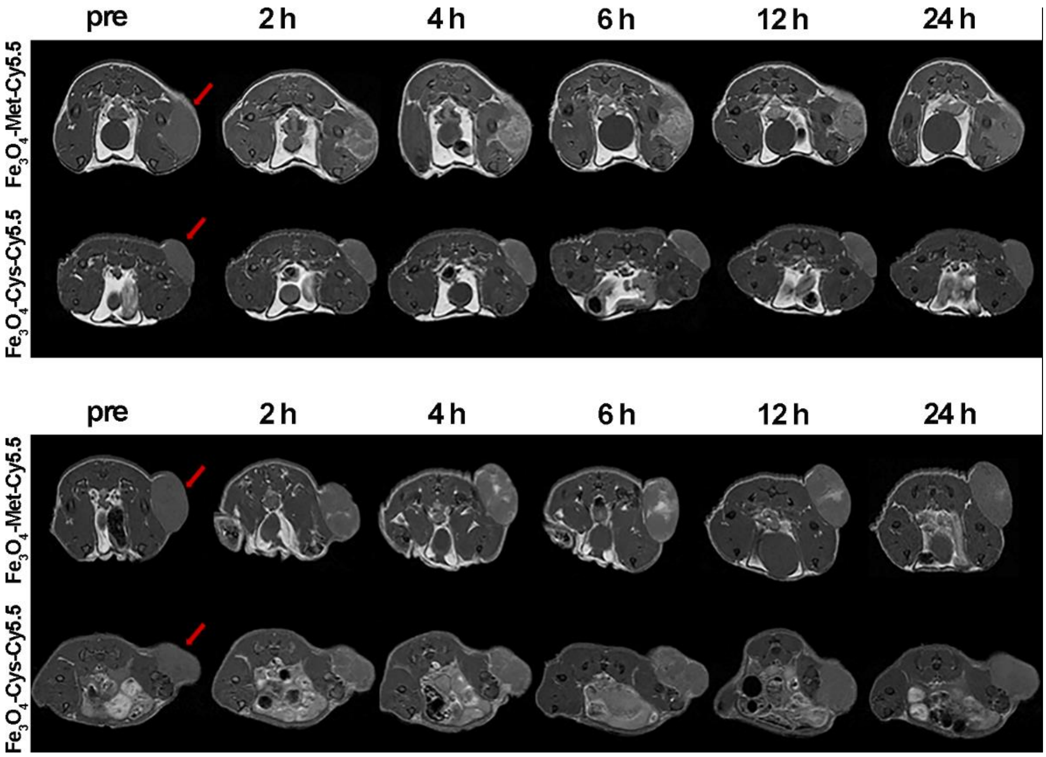

Figure S7. T_1-weighted MRI of two additional tumor-bearing mice for each group within 24 h before and after injection with Fe_3O_4-Met-Cy5.5 or Fe_3O_4-Cys-Cy5.5.

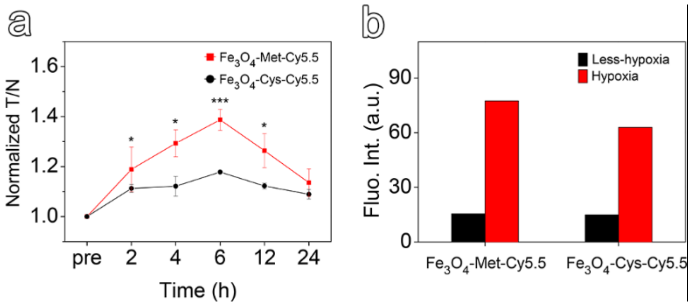

Figure S8. (**a**) The trend of MRI signal at different time points in tumors injected with Fe_3O_4-Met-Cy5.5 or Fe_3O_4-Cys-Cy5.5. (**b**) Semi-quantitative analysis of HIF-1α expression of the less-hypoxic and hypoxic regions in tumors treated with Fe_3O_4-Met-Cy5.5 or Fe_3O_4-Cys-Cy5.5. *$p < 0.05$, ***$p < 0.001$.

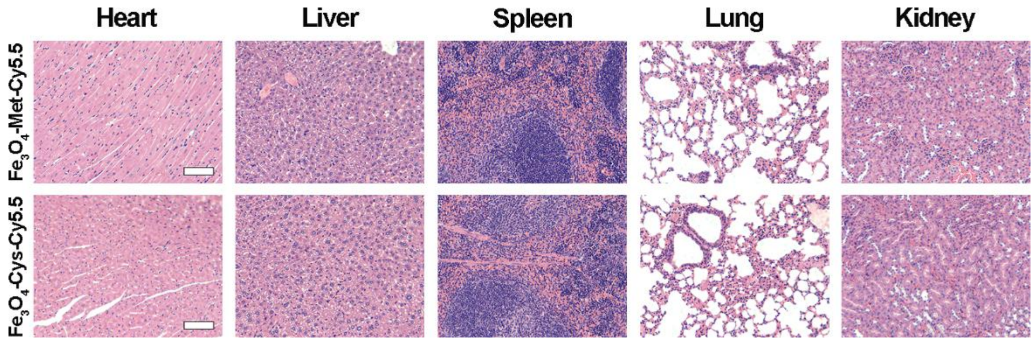

Figure S9. H&E staining of organ tissue (heart, liver, spleen, lung, and kidney) from mice harvested at 24 h after the injection of Fe_3O_4-Met-Cy5.5 or Fe_3O_4-Cys-Cy5.5 (scale bar represents 100 μm).

References

1. Guo, S.; Sun, D.; Ni, D.; Yu, M.; Qian, K.; Zhang, W.; Yang, Y.; Song, S.; Li, Y.; Xi, Z.; Wang, J.; Li, J. y.; Wei, Y.; Chen, K.; Gan, Y.; Wang, Z., Smart Tumor Microenvironment-Responsive Nanotheranostic Agent for Effective Cancer Therapy. *Adv. Funct. Mater.* **2020**, *30* (17), 2000486.
